# Behavioral and biochemical evidence of the role of acetaldehyde in the motivational effects of ethanol

**DOI:** 10.3389/fnbeh.2013.00086

**Published:** 2013-07-15

**Authors:** Alessandra T. Peana, Elio Acquas

**Affiliations:** ^1^Department of Chemistry and Pharmacy, University of SassariSassari, Italy; ^2^Department of Life and Environmental Sciences - Pharmaceutical, Pharmacological and Nutraceutical Sciences Section, University of CagliariCagliari, Italy

**Keywords:** acetaldehyde, behavior, ethanol, dopaminergic transmission, extracellular signal regulated kinase, opioidergic transmission, salsolinol

## Abstract

Since Chevens' report, in the early 50's that his patients under treatment with the aldehyde dehydrogenase inhibitor, antabuse, could experience beneficial effects when drinking small volumes of alcoholic beverages, the role of acetaldehyde (ACD) in the effects of ethanol has been thoroughly investigated on pre-clinical grounds. Thus, after more than 25 years of intense research, a large number of studies have been published on the motivational properties of ACD itself as well as on the role that ethanol-derived ACD plays in the effects of ethanol. Accordingly, in particular with respect to the motivational properties of ethanol, these studies were developed following two main strategies: on one hand, were aimed to challenge the suggestion that also ACD may exert motivational properties on its own, while, on the other, with the aid of enzymatic manipulations or ACD inactivation, were aimed to test the hypothesis that ethanol-derived ACD might have a role in ethanol motivational effects. Furthermore, recent evidence significantly contributed to highlight, as possible mechanisms of action of ACD, its ability to commit either dopaminergic and opioidergic transmission as well as to activate the Extracellular signal Regulated Kinase cascade transduction pathway in reward-related brain structures. In conclusion, and despite the observation that ACD seems also to have inherited the elusive nature of its parent compound, the behavioral and biochemical evidence reviewed points to ACD as a neuroactive molecule able, on its own and as ethanol metabolite, to exert motivational effects.

## Introduction

Acetaldehyde (ACD) is well-known as a toxic compound and on this property was grounded the rationale for the use of disulfiram, an aldehyde dehydrogenase inhibitor, to treat alcoholism. However, toxicity does not necessarily involve perceived aversive effects and at the basis of this treatment are its aversive effects (nausea, headache, hot flushes, etc.) that discourage consumption. In spite of this, however, in the early 50's it was reported that some patients under treatment with the aldehyde dehydrogenase inhibitor (ALDH), antabuse, could experience pleasurable effects while ingesting small amounts of ethanol and it was postulated that ACD might have positive emotional as well as motivational effects (Chevens, 1953). After a long period of obsolescence from this observation, in the last decades the research on the role of ACD in the effects of ethanol, both as ethanol's metabolite and as chemical with its own motivational properties, has seen a renewed interest. To address this issue, the first approach has been to consider ethanol as a pro-drug of ACD. Indeed, the most radical view suggested that ACD could be responsible for all of the effects of ethanol and that *alcoholism* might, instead, be termed *acetaldehydism* (Raskin, 1975). Notably, a consistent body of evidence, suggesting that to exert its motivational properties ethanol must be metabolized into ACD, has been collected by different approaches including catalase manipulations (Aragon et al., 1985, 1991; Aragon and Amit, 1992), the use of alcohol dehydrogenase (ADH) (Amit, 1977; Brown et al., 1979; Smith et al., 1984; Quertemont and De Witte, 2001; Peana et al., 2008a) or ALDH inhibitors (Amit, 1977; Spivak et al., 1987a,b; Suh et al., 2006), the use of knock-out mice for the CYP2E1 isoform (Suh et al., 2006; Correa et al., 2009a) and the use of lentiviral vectors to silence the cell genome encoding for catalase or ADH synthesis (Karahanian et al., 2011). These approaches generated a large number of studies, summarized in comprehensive reviews (Quertemont et al., 2005; Correa et al., 2012), showing that locomotor (Escarabajal and Aragon, 2002; Martí-Prats et al., 2010; Ledesma and Aragon, 2012), anxiolytic (Correa et al., 2008; Escrig et al., 2012) and, in particular, motivational (Peana et al., 2008a,b, 2009, 2010a) properties of ethanol could be prevented by inhibiting either its peripheral and central metabolism or by ACD inactivation. Notably, two further issues, one related to the questioned ability of ACD to cross the blood brain barrier [see Correa et al. (2012) for an extensive discussion on this issue] and another related to the role of enhanced ethanol plasma concentrations that may in turn reach the brain, require to be dealt with while taking into consideration the consequences of blockade of ethanol peripheral metabolism.

Another approach to address the role of ACD in the motivational properties of ethanol has been to consider it as a chemical with neurobiological properties on its own. Indeed, also this line of investigation has generated a significant body of data that also converged toward the characterization of ACD as a neurochemical agent able to elicit locomotor activity (Correa et al., 2009b) and anxiolytic effects (Correa et al., 2008), to sustain drug discrimination (York, 1981; Redila et al., 2000, 2002; Quertemont and Grant, 2002), to affect cognition (Sershen et al., 2009), and to elicit motivational effects (York, 1981; Peana et al., 2008a, 2009, 2010b; Spina et al., 2010). Interestingly, the behavioral evidence for the characterization of ACD as a drug with motivational properties was gathered, from conditioned place preference (CPP) and self-administration studies, in parallel with electrophysiological, biochemical and immunohistochemical studies pointing also to the critical role of dopamine (DA) (Foddai et al., 2004; Melis et al., 2007; Enrico et al., 2009; Spina et al., 2010; Vinci et al., 2010; Sirca et al., 2011) and opioid (Pastor et al., 2004; Sánchez-Catalán et al., 2009; Peana et al., 2011) transmission as well as to the involvement of Extracellular signal Regulated Kinase (ERK) (Spina et al., 2010; Vinci et al., 2010) at the basis of ACD's motivational properties. The present review aims to recapitulate this evidence in support of the tenet of ACD as a molecule able to exert motivational effects in rodents (for a recent comprehensive review see Correa et al., 2012).

## Conditioned place preference and self-administration studies

The role of ACD in the positive motivational properties of ethanol has become an increasingly attractive matter of debate and many studies have attempted to establish whether ACD is necessary for the manifestation of the neurobiological and behavioral effects of ethanol. Such studies have been developed by the aid of compounds that increase as well as inhibit, both centrally and peripherally, the formation of ACD and by the aid of compounds able to sequester ACD into stable non-reactive adducts. ACD has been shown to elicit CPP after intracerebroventricular infusion (Smith et al., 1984) and after intragastric (Peana et al., 2008a) and intraperitoneal (Quertemont and De Witte, 2001) administration. Notably, under these conditions, ACD-elicited CPP shows a bell shaped dose-response curve similar to that of ethanol (Quertemont and De Witte, 2001; Peana et al., 2008a).

The isoforms of ADH, normally found in gastric and hepatic tissue, represent the main metabolic pathway by which ethanol is converted into ACD upon ingestion (Baraona et al., 1991). Hence, provided that ethanol is not a substrate of brain ADH isoforms, the effect of the ADH competitive inhibitor, 4-methylpyrazole (4-MP), mostly used in behavioral studies is restricted to the peripheral metabolism of ethanol (Escarabajal and Aragon, 2002). In this paragraph we will focus our attention mostly on studies in which, to mimic the route commonly used by humans, ethanol and ACD were administered orally. In a CPP study, we showed that pretreatment with 4-MP reduces intragastric ethanol- (1 g/kg) but not ACD- (20 mg/kg) induced CPP, suggesting that ACD, metabolically derived from ethanol, could be responsible for this effect (Peana et al., 2008a). As previously mentioned, however, the impact of these results is partly reduced by the observation that administration of 4-MP may also affect, indirectly, brain ACD (Spivak et al., 1987a,b). In fact, when ethanol's metabolic conversion is prevented in the periphery it can reach the brain in greater amounts and can be metabolized therein by alternative pathways (catalase) (Aragon et al., 1985, 1991). This observation is also in agreement with the study by Tambour et al. (2007) reporting that the administration of cyanamide, a catalase and ALDH inhibitor, could prevent the locomotor stimulant effects of ethanol in mice and that this effect could be prevented by previous administration of 4-MP (Sanchis-Segura et al., 1999; Tambour et al., 2007).

Evidence of the role of ACD in the motivational properties of ethanol has also been provided by CPP experiments with D-penicillamine, a compound, which acts by sequestering ACD into a non-reactive and stable adduct without altering ethanol metabolism (Nagasawa et al., 1978). Thus, peripheral administration of D-penicillamine, at doses devoid of motivational properties *per se*, prevents the acquisition of either ethanol- (Font et al., 2006a; Peana et al., 2008a) or ACD-induced CPP (Peana et al., 2008a). In addition, as in the case of the experiments with 4-MP, the specificity of pretreatment with D-penicillamine on ethanol-derived ACD was confirmed by its failure to affect morphine-elicited CPP. Further, indirect, evidence of the role of ACD in the motivational effects of ethanol comes from studies with L-cysteine, a thiol amino acid, also known for its ability to protect against ACD toxicity (Salaspuro, 2007). L-cysteine is a precursor of the antioxidant glutathione (Soghier and Brion, 2006) and binds ACD by way of cysteinylglycine, the first metabolite in glutathione breakdown (Kera et al., 1985). Notably, L-cysteine formulations have been developed to bind in the oral cavity ACD originating after heavy smoking and alcohol drinking (Salaspuro et al., 2002; Salaspuro, 2007). Such formulations offer a novel method for intervention studies aimed to fight the role of ACD in the pathogenesis of upper digestive tract cancer (Salaspuro, 2007), alcoholic cardiomyopathy, as well as against the chronic toxicity of ACD (Sprince et al., 1974). Accordingly, we found that L-cysteine reduces either ethanol- and ACD-elicited CPP (Peana et al., 2009) supporting the notion that the generation and accumulation of ACD actively contributes to ethanol-induced CPP.

Several studies have found that ACD supports its self-administration. This has been demonstrated in unselected rats self-administering ACD intravenously (Myers et al., 1982; Takayama and Uyeno, 1985), intracerebroventricularly (Brown et al., 1979) and into the VTA (McBride et al., 2002), the latter also in alcohol-preferring rats (Rodd-Henricks et al., 2002). Further evidence that ACD exerts positive motivational properties arises from the observation that rats also acquire and maintain oral ACD self-administration (Peana et al., 2010b; Cacace et al., 2012). Our work, in this regard, has been aimed to characterize the role that ethanol-derived ACD plays in the pharmacological properties of orally ingested ethanol. Thus, as in the CPP studies, we have found that inhibition of the metabolism of ethanol, by 4-MP, reduces oral ethanol self-administration behavior (Peana et al., 2008b) an effect that might also be attributed to an increased concentration of ethanol which falls on the right-hand side of its bell shaped dose response curve. On the other hand, we have recently demonstrated, for the first time that ACD shares with ethanol the ability to induce oral self-administration behavior further supporting the hypothesis that ACD itself exerts motivational effects (Peana et al., 2010b). Notably, rats self-administering ACD show extinction behavior when ACD is discontinued while gradually reinstate operant responses when ACD is reintroduced (Peana et al., 2010b). In addition, studies directed toward the characterization of the role of ACD, provided evidence that ethanol-derived ACD plays an important role in ethanol's motivational effects. Accordingly, previous findings have reported that intracerebroventricular the structural analogue of L-cysteine, D-penicillamine, reduces voluntary ethanol consumption in rats indicating that the central inactivation of ACD also blocks ethanol intake (Font et al., 2006b). On the other hand, L-cysteine reduces the acquisition and maintenance of oral ethanol self-administration as well as the reinstatement of ethanol-drinking and ethanol-seeking behaviors (Peana et al., 2010a, 2013a). Others studies examined the motivational effects of ACD under the break point that serves as an index of animals' motivation to work for the reinforcer. In these experiments 0.2% v/v ACD's break point was not statistically different from the break point of 10% v/v ethanol in spite of a 50 times lower concentration (Peana et al., 2012). On the other hand, ACD consumatory responses were paralleled by a relevant increase in ACD blood but not brain concentrations (Peana et al., 2010b). Furthermore, as in CPP and ethanol self-administration studies, during oral ACD self-administration L-cysteine was shown to decrease acquisition, maintenance, deprivation effect as well as ACD break point without interfering with saccharin reinforcement (Peana et al., 2012).

The ability of L-cysteine to affect ethanol-induced motivation could reside in different mechanisms. The first is consistent with the conjugation/inactivation mechanism that would take place between ACD and the first metabolite of glutathione (Kera et al., 1985). Furthermore, being L-cysteine an analogue of L-glutamate (Thompson and Kilpatrick, 1996) it is posited to interact at presynaptic group I metabotropic glutamate receptors (mGluR) of the mGluR5 subtype to exert a positive modulatory control on synaptic glutamate release (Harman et al., 1984; Croucher et al., 2001). In support of these results is the finding that L-cysteine reduces ethanol-induced stimulation of DA transmission in the nucleus accumbens shell (Sirca et al., 2011). Finally, it is noteworthy in this regard that L-cysteine may cross the blood brain barrier through excitatory amino acid transporters (Chen and Swanson, 2003) thus leaving open the possibility that this compound might also act centrally.

The main system of central ethanol oxidation is mediated by the enzyme catalase (Aragon et al., 1991; Aragon and Amit, 1992; Zimatkin et al., 1998). By reacting with H_2_O_2_, brain catalase forms compound I (the catalase-H_2_O_2_ system), which is able to oxidize ethanol into ACD (Pastor et al., 2002; Ledesma and Aragon, 2012). Recently we showed that the H_2_O_2_ scavenging agent, alpha lipoic acid, dose-dependently reduces the maintenance and break point of oral ethanol self-administration under a progressive ratio schedule as well as the reinstatement of ethanol seeking behavior without suppressing saccharin self-administration (Peana et al., 2013b). On a similar vein, a recent study by Ledesma and Aragon (2013) demonstrated that alpha lipoic acid reduces the acquisition and reconditioning of ethanol-induced CPP in mice. Overall, these data support the suggestion that a decrease in cerebral H_2_O_2_ availability, i.e., a reduced metabolic activity of brain catalase, by alpha lipoic acid administration may inhibit oral ethanol self-administration further suggesting that the brain catalase-H_2_O_2_ system, and therefore centrally formed ACD, plays a key role in the motivational effects of ethanol.

## Acetaldehyde, dopamine, and intracellular signaling

The acute administration of ethanol elicits DA transmission in the rat nucleus accumbens (Imperato and Di Chiara, 1986; Howard et al., 2008) and this is also true in men whereby these increases in the ventral striatum positively correlate with the psychostimulant effect of ethanol (Boileau et al., 2003). Thus, a physiologically active DA transmission may represent the prerequisite for ethanol and, in accordance with Chevens (1953), also for ACD being able to elicit motivational effects. In this regard, in light of the hypothesized role of DA in the motivational effects of ACD, an indirect support comes from the clinical observation that subjects administered the DA biosynthesis inhibitor, alpha-methyltyrosine, do not experience ethanol-induced stimulation and euphoria (Ahlenius et al., 1973). With these premises in mind this paragraph will present and discuss the preclinical studies that support the involvement of DA in the motivational effects of ACD.

The main line of experimental evidence of the involvement of DA in the effects of ACD was inspired by the known ability of ethanol to stimulate the firing rate of midbrain DA cells either *in vivo* (Gessa et al., 1985; Foddai et al., 2004) and *in vitro* (Brodie et al., 1990; Melis et al., 2007) as well as the release of DA from mesoaccumbens terminals (Imperato and Di Chiara, 1986; Howard et al., 2008). Indeed these data also inspired the experiments aimed to test whether ethanol-derived ACD, as well as ACD on its own, could stimulate the firing rate of DA neurons in the VTA (Foddai et al., 2004) and DA transmission in the nucleus accumbens (Ward et al., 1997; Melis et al., 2007; Enrico et al., 2009). Thus, in keeping with the well-established paradigm, successfully applied in behavioral (Peana et al., 2008a) and histochemical (Vinci et al., 2010) studies that envisioned the use of 4-MP to prevent ethanol peripheral metabolism, Foddai et al. (2004) showed that this ADH inhibitor prevents ethanol-elicited stimulation of firing rate of DA neurons in the VTA, demonstrating for the first time that metabolic conversion of ethanol into ACD plays a critical role in the ability of ethanol to activate mesencephalic DA cells (Foddai et al., 2004). This finding has been further extended by the study of Melis et al. (2007) in which, by recording VTA DA cells *ex vivo*, it was shown that ethanol and ACD similarly stimulate DA cells firing rate but also that the stimulatory effect of ethanol is prevented by inhibition of its metabolic conversion into ACD by the catalase inhibitor 3-aminotriazole (Melis et al., 2007).

Notably, all the above refers to the ability of ACD to affect DA cells function and transmission at the pre-synaptic level. Immunohistochemical data, however, have shown that the expression of phosphorylated Extracellular signal regulated kinase (pERK), taken as an index of post-synaptic DA-dependent neuronal activation (Acquas et al., 2007; Ibba et al., 2009), is similarly increased in the nucleus accumbens after the acute oral administration of either ethanol (Ibba et al., 2009; Vinci et al., 2010) and ACD (Vinci et al., 2010). In addition, ethanol-elicited increase of pERK expression in the nucleus accumbens could be prevented either by 4-MP and D-penicillamine (Vinci et al., 2010). ERK phosphorylation may take place by a number of factors ranging from extracellular signals to increased intracellular Ca^++^ concentrations via the sequential activation of a kinase cascade (Sweatt, 2004). This activated kinase has been related to neuronal plasticity (Fasano and Brambilla, 2011) and to long-term behavioral events that may be triggered by addictive drugs (Valjent et al., 2004; Girault et al., 2007) such as acquisition of conditioned responses (Beninger and Gerdjikov, 2004) as well as reinstatement of ethanol seeking (Radwanska et al., 2008; Schroeder et al., 2008; Peana et al., 2013a) in self-administration experiments. Accordingly, we found that while acute ACD administration elicits pERK in the nucleus accumbens (and other nuclei of the extended amygdala) (Vinci et al., 2010), the intracerebroventricular administration of PD98059, an inhibitor of the mitogen-activating extracellular kinase (MEK), the kinase responsible of ERK phosphorylation, prevents the acquisition of ACD-elicited CPP (Spina et al., 2010). In addition, the involvement of DA in these studies is supported by the experiments with the DA D_1_ antagonist SCH 39166 (Ibba et al., 2009; Spina et al., 2010; Vinci et al., 2010). In fact, we demonstrated that either oral ethanol- (Ibba et al., 2009) and ACD- (Vinci et al., 2010) elicited nucleus accumbens increases of pERK expression and ACD-elicited CPP (Spina et al., 2010) could be prevented by blockade of D_1_ receptors by SCH 39166.

A further line of experimental evidence, though yet speculative, of the involvement of DA in the effects of ACD originates from the studies on the condensation product(s) of ACD which, in particular when condensates with DA, can either spontaneously and enzymatically (Chen et al., 2011) generate 1-methyl-6,7-dihydroxy-1,2,3,4-tetrahydroisoquinoline (salsolinol) (Yamanaka et al., 1970; Jamal et al., 2003). Interestingly, since its discovery, this molecule has been related to ethanol (Davis and Walsh, 1970; Davis et al., 1970) and it was hypothesized that at least the DA-mediated effects of ethanol could be attributed to salsolinol (Davis and Walsh, 1970; Davis et al., 1970). This compound, indeed, has recently been reported to stimulate *in vitro* DA cells firing rate in the posterior VTA (Xie and Ye, 2012; Xie et al., 2012) and also, when applied into the VTA *in vivo*, to elicit DA transmission in the rat nucleus accumbens (Rodd et al., 2003; Deehan et al., 2012) and sustain its self-administration (Rodd et al., 2008). Thus, these data seem to collectively point to salsolinol as a substance that may exert motivational effects by virtue of its ability to involve mesolimbic DA. However, the relationship between ACD, DA and salsolinol still awaits to be fully disclosed.

## Acetaldehyde and opioids

Acute administration of ethanol increases endogenous opioid (endorphin and enkephalin) release from brain slices, pituitary gland and increases blood levels of opioids in humans *in vivo* (see Herz, 1997 for a comprehensive review). Acute alcohol administration also enhances gene expression of both endorphin and enkephalin in selected brain areas of rats, whereas chronic ethanol administration reduces gene expression, making less of opioid peptides available for release (Herz, 1997). Since opioid transmission, in both the VTA and nucleus accumbens, regulates the release of DA from mesolimbic neurons, ethanol-induced opioid release may produce reinforcement by modulating DA transmission. Accordingly, opioid antagonists decrease the motivational properties of ethanol in rats self-administering ethanol by interfering with ethanol-dependent dopaminergic activation (Acquas et al., 1993; Benjamin et al., 1993; Di Chiara et al., 1996; Gonzales and Weiss, 1998) as the motivational effects of ethanol by opioid antagonists may involve an opioid-DA link (Di Chiara et al., 1996). However, another line of evidence for the involvement of the endogenous opioid system in the motivational effects of ethanol appears to be related to its first metabolite, ACD. In fact, there are several proofs to support that opioids are implicated in the motivational effects of ACD as well. The first arises from the early observation by Myers et al. (1984) demonstrating that naloxone decreases intravenous ACD self-administration. The second arises from the observation that naltrexone reduces oral ACD self-administration (Peana et al., 2011). In particular, we observed that this effect could be mediated by an involvement of μ_1_ opioid receptors since naloxonazine, a μ_1_ selective opioid receptor antagonist, reduced the maintenance phase of ACD self-administration (Peana et al., 2011). The mechanism by which opioid antagonists affect the motivational properties of ACD is unclear. In this regard one might consider that ACD on its own, and as a consequence of the metabolism of ethanol, activates neuronal firing of DA cells in the VTA (Foddai et al., 2004), stimulates DA transmission (Melis et al., 2007; Enrico et al., 2009; Sirca et al., 2011) and ERK phosphorylation in the nucleus accumbens (Vinci et al., 2010). This possibility appears in agreement with the observation that blockade of μ opioid receptors also prevents ERK phosphorylation in the nucleus accumbens (Peana et al., 2011). An alternative possibility, yet to be fully demonstrated, is offered by the suggestion that the condensation product between ACD and DA, salsolinol (see above), acts *via* stimulation of μ opioid receptors (Hipolito et al., 2009, 2010, 2012; Xie et al., 2012).

## Discussion and conclusions

The reviewed literature indicates that ACD has its own motivational properties as assessed by CPP and self-administration studies (Peana et al., 2008a, 2010b; Spina et al., 2010) and also that this property is grounded on its ability to involve mesolimbic DA (Foddai et al., 2004; Melis et al., 2007; Spina et al., 2010; Vinci et al., 2010), μ opioid receptors -mediated transmission (Peana et al., 2011) as well as phosphorylated ERK in the nucleus accumbens (Spina et al., 2010; Vinci et al., 2010; Peana et al., 2011). In addition, a wealth of experimental evidence supports that the motivational effects of ethanol are mediated by its metabolism into ACD either in the periphery or in the brain. Accordingly, this has been demonstrated by inhibiting the production of ACD in the periphery (inhibition of ADH), by inhibiting the generation of brain ACD (inhibition of brain catalase) or by reducing ACD bioavailability (Font et al., 2006a,b; Peana et al., 2008a, 2009, 2010a, 2013a,b; Enrico et al., 2009; Sirca et al., 2011; Correa et al., 2012). All these observations support the tenet that the generation of central and peripheral, but not peripherally accumulated (Escrig et al., 2012), ACD actively participates in the positive motivational properties of ethanol and raise the possibility that its role can be exploited to devise novel pharmacological approaches that target alcohol abuse related problems.

### Conflict of interest statement

The authors declare that the research was conducted in the absence of any commercial or financial relationships that could be construed as a potential conflict of interest.
